# Assessment of mental distress among prison inmates in Ghana’s correctional system: a cross-sectional study using the Kessler Psychological Distress Scale

**DOI:** 10.1186/s13033-015-0011-0

**Published:** 2015-03-29

**Authors:** Abdallah Ibrahim, Reuben K Esena, Moses Aikins, Anne Marie O’Keefe, Mary M McKay

**Affiliations:** School of Public Health, College of Health Sciences, University of Ghana, Accra, Ghana; School of Community Health and Policy, Morgan State University, Baltimore, MD USA; Global Institute of Public Health, New York University, New York, NY USA

**Keywords:** Mental health, Ghana prisons, Northern Ghana, Kessler Psychological Distress Scale

## Abstract

**Background:**

Applying global estimates of the prevalence of mental disorders suggests that about 2.4 million Ghanaians have some form of psychiatric distress. Despite the facts that relatively little community-based treatment is available (only 18 psychiatrists are known to actively practice in Ghana), and that mental disorders are more concentrated among the incarcerated, there is no known research on mental disorders in Ghana prisons, and no forensic mental health services available to those who suffer from them. This study sought to determine the rate of mental distress among prisoners in Ghana.

**Methods:**

This cross-sectional research used the Kessler Psychological Distress Scale to estimate the rates and severity of non-specific psychological distress among a stratified probability sample of 89 male and 11 female prisoners in one of the oldest correctional facilities in the country. Fisher’s exact test was used to determine the rates of psychological distress within the study population.

**Results:**

According to the Kessler Scale, more than half of all respondents had moderate to severe mental distress in the four weeks preceding their interviews. Nearly 70% of inmates with only a primary education had moderate to severe mental distress. Though this was higher than the rates among inmates with more education, it exceeded the rates for those with no education.

**Conclusions:**

The high rate of moderate to severe mental distress among the inmates in this exploratory study should serve as baseline for further studies into mental disorders among the incarcerated persons in Ghana. Future research should use larger samples, include more prison facilities, and incorporate tools that can identify specific mental disorders.

## Background

Every year millions of people worldwide are diagnosed with one or more disabling mental disorders. Unfortunately, mental disorders, which are responsible for increasing medical care costs and lost productivity every year, do not get the same attention that physical illnesses do. It is estimated that globally, mental disorders account for 12% of the burden of diseases, and this is expected to increase to 15% by 2020 [1]. However, among the adult population alone in Ghana, mental disorders account for 13% of the nation’s burden of disease [2]. The World Health Organization (WHO) also estimates that worldwide, about 25% of a country’s population will suffer from a mental disorder at some point in their lives. At any point in time, an estimated 10% of people suffer from a psychiatric condition, including an estimated one percent who have a severe form of mental disorder [2,3]. Applying the WHO global estimates to Ghana’s most recent population census suggests that about 2.4 million Ghanaians may have some form of psychiatric condition [2]. More recently, the World Health Organization has estimated that about 650,000 Ghanaians (or 3% of the population) have a severe form of mental disorder [2].

With only 18 psychiatrists known to be actively practicing in Ghana and an estimated 2.4 million Ghanaians suffering from at least one mental disorder, there is a significant gap between need and the availability of mental health services in the country [2-4]. Despite the incredible unmet need for mental health care, little emphasis has been put on this evolving crisis in Ghana’s public mental health system.

On average, more than 6,300 individuals are admitted annually to the state psychiatric facilities that exist in Ghana, all of them located in the urban parts of the country [2,3]. The primary psychiatric diagnoses for these admissions are schizophrenia, depression, and substance use disorder [3]. Many other individuals who are believed to be experiencing a form of mental disorder are sent or referred to non-traditional healing or treatment facilities known as “prayer camps”, or to other alternative African traditional healing centers. Because of the limited number of public psychiatric facilities and the large number of people who need treatment, the non-traditional treatment centers see the majority of patients who need care in Ghana.

In addition to the people in mental health institutions and those accessing the prayer camps for mental health care, there are a growing number of people in Ghana in the criminal justice system who are believed to be suffering from many forms of mental disorders [4]. Since there is little or no pressure to address this problem among the general Ghanaian population, those with mental distress who are incarcerated have little recourse.

Research has estimated that between 16% and 64% of individuals who are incarcerated or have a history of involvement in the criminal justice system suffer from mental disorders [5-7]. Although there is no known research on the number of incarcerated Ghanaians with mental disorders, the true prevalence may be similar to what has been found in other African countries including South Africa, Zambia, and Nigeria [7-9].

Having a mental disorder by itself is not a crime. However, mental illness may influence a person to commit a crime and therefore lead to incarceration. The colonial legacy approach in Ghana reveals how little is understood about treatments that could enable individuals with mental disabilities to live normal, integrated, and productive lives in their communities. Forensic mental health services, i.e., the interface between mental disorders and the criminal justice system, have not been studied and are not addressed by the new Mental Health Act (MHA) enacted by Ghana’s Parliament in 2012 [1]. The MHA of 2012 replaced the archaic Mental Health Decree of 1972 that had emphasized institutionalizing individuals with mental disorders [3]. The MHA seeks to reorient the country’s response to mental disorders toward non-institutional treatment.

Although the new mental health law is an important step to adequately addressing the country’s mental health needs, little has been done to implement it. In fact, very little is known about the true prevalence of mental disorders within the general population, let alone among those in Ghana’s correctional system. The few studies of mental disorders in prison or jail populations that have been done in Africa were conducted in South Africa, Zambia, Nigeria, and a few other African countries [7-10].

To address the gaps in our knowledge, this exploratory study sought to determine the rates of mental distress among those involved in the correctional system in Ghana using a validated screening tool.

## Methods

This cross-sectional research study used the Kessler Psychological Distress Scale (K10) (interviewer administered version) developed for use in the United States’ National Health Interview Survey to estimate the prevalence of mental distress [11]. The interviewer administered version was used because of the high rates of illiteracy in Northern Ghana and among the population studied. Scores on the K10 range from 10 to 50, corresponding to the severity of a respondent’s mental distress. If an individual scores under 20 on the Kessler tool, it reflects relatively good mental health; scores of 20–24 reflect a mild mental disorder; individuals with scores of 25–29 are likely to have a moderate mental disorder; and individuals who score 30 to 50 are likely to have a severe mental disorder [11].

The Kessler instrument has been used in several countries including South Africa and Nigeria, where the validity of the instrument was established for Africa [12]. The tool has also been used in Australia to assess the level of psychological distress among prisoners in the Australian National Prisoner Health Census [13].

The K10 tool is a brief, client-friendly screener. It is much easier to administer than more extensive screening tools such as the World Mental Health Composite International Diagnostic Interview (WMH-CIDI) that requires interviewers to undergo mandatory training available only at WHO-authorized Training and Reference Centers. The K10 is also more appropriate for this research than the patient health questionnaire (PHQ-9) and mood disorder questionnaire (MDQ) that are tailored for specific mental disorders.

### Study setting

The participants interviewed for this study were serving time at the Tamale central correctional facility in the Northern Region of Ghana, the largest of Ghana’s ten administrative regions. This facility was chosen because it is one of the oldest prison facilities in Ghana’s correctional system. The interview process included a demographic data questionnaire in addition to the main Kessler screening instrument.

Tamale is the fourth largest city in Ghana. It has a population of less than 400,000 (50% are female and 50% male), and an annual population growth rate of about three percent [14]. The Tamale central prison facility houses an inmate population including individuals serving life sentences, terminal sentences, and those being held for pre-trial detention. The inmates in the facility consist of various ethnic tribes, including those from the Northern Region such as the Mossi, Dagomba, Nanumba, Mamprusi, and Gonja that are collectively called the Mole-Dagomba ethnic group. There are many other ethnic tribes in Northern Ghana that are not part of the Mole-Dagomba ethnic group and are mainly located in the north-east and north-west parts of Northern Ghana. Other ethnic tribes imprisoned in the Tamale correctional facility include tribes such as the Akan, Ga-Adangbe, and Ewe among others. These tribes are mainly located in the southern part of Ghana and are collectively referred to here as Southern-tribes.

Similar to the other correctional facilities in Ghana, the prison in Tamale faces problems including aging structures, overcrowding, and a lack of adequate sanitation. All together, correctional facilities in Ghana house approximately 14,000 individuals [15].

### Sampling

The prison facility had an inmate population of 280 at the time of the interviews, including 267 men and 13 women. A stratified probability sampling method was used to select the respondents for the study. The facility inmate population was first stratified by sex and the men were sampled under a simple random sampling method. The rest of the men who could provide written informed consent were not interviewed because the study did not aim to penetrate the entire population. Since the facility was holding only 13 women at the time of interviews, all but two who did not provide a written consent to participate were interviewed. The total sample used for this study was 100 respondents, including 89 men and 11 women.

### Statistical analysis

The study’s statistical analysis was done using Stata, Version 11.2 (Stata Corp, College Station, TX). Fisher’s exact test was used to determine the proportion of mental distress among the sampled inmate population. The Fisher’s exact test was used because some of the variables in the sample included cells with less than five individuals. Analyses with a p-value ≤ 0.05 were considered statistically significant.

### Ethical considerations

Because the study involved an incarcerated population, the nature and scope of the research was explained to each of the inmates before the interview began. Prisoners are considered vulnerable because they are involuntarily institutionalized and might be prone to coercion and undue influence. Those who were randomly selected for the face-to-face interviews were provided information about the study and a written informed consent document to help them freely decide whether or not to participate. Each inmate was informed that the Ghana Prisons authority was not requiring anyone to participate in the interviews, and each inmate was assured that there would be no retribution for refusing to participate. All the sampled inmates who were interviewed provided written informed consent and had the opportunity to ask questions before the interview. They were also advised that any information provided during their face-to-face interview would remain confidential.

The study was approved by the University of Ghana’s Ethical Committee for the Humanities (ECH). The Ghana Prisons Service national headquarters in Accra and the Regional Prison Command in Tamale also authorized the study. Interviews were conducted in a comfortable room inside the facility.

## Results

A total of 100 inmates, including 89 men and 11 women, were interviewed for this study. Table 1 displays the socio-demographic characteristics of the 100 individuals included in the study. The mean age was 37 years old with a standard deviation of 15.7 years. The youngest inmates were an 18 year-old male and female; the oldest was a 101-year-old man. A majority (51%) of the respondents were between the ages of 30 and 59. Most of the inmates were either Northern Ghana’s Mole-Dagomba ethnic group (38%) or belonged to other collective minor tribes (39%) in Northern Ghana. Fifty percent of the respondents had less than primary or no formal education. More than half of the inmates indicated they were of Moslem faith (54%). Of the total sample, 65% were registered for Ghana’s National Health Insurance Scheme (NHIS).

**Table 1:** **Socio-demographic characteristics of 100 incarcerated individuals at the Ghana Prisons Service in the Northern Region**

**Variables (N = 100)**	**Percentage**
**Age**	
<30	38
30-59	51
60-101	11
**Sex**	
Male	89
Female	11
**Education**	
Primary	23
Secondary	25
College	2
None	50
**Ethnicity**	
Southern tribes	23
Northern Mole-Dagomba	38
Other - Northern tribes	39
**Religion**	
Moslem	54
Christian	46
**NHIS**	
Yes	65
No	35

*Note*: The “Mole-Dagomba” is an ethnic group that consists of the Mossi, Dagomba, Nanumba, Mamprusi, and Gonja tribes in the Northern Region. The “Other” ethnic group includes various tribes from the other regions in the northern part of Ghana. The “Southern tribes” ethnic group includes the Akan, Ga-Adangbe and Ewe tribes.

Table 2 shows the study prevalence of mental distress among the sampled incarcerated individuals distributed according to the Kessler Psychological Distress Scale (K10) categories. A majority of the respondents in all the age categories had moderate to severe mental distress scores in the four weeks preceding the interviews, according to the Kessler scale. Overall, more than half of all the respondents had a K10 score of 25 or higher (moderate to severe mental distress). Sixty percent of those without formal education had K10 scores of 25–50, which put them in the moderate to severe mental distress category. More than one-third of prisoners with or without NHIS registration had moderate mental distress. Twenty-six percent of those with NHIS registration had scores that put them in the severe mental distress category compared to 34% of prisoners without NHIS registration who had severe mental distress. More than one-third of the Moslem respondents were classified as having severe mental distress. More than one-third of all male respondents (33.7%) and 45.5% of the females had moderate mental distress.

**Table 2:** **Study rates of mental distress among incarcerated individuals in Ghana’s Prison Service by Kessler Psychological Distress Scale and by selected demographic variables**

	**Kessler Psychological Distress Score (K10)**				
	**0 - 19**	**20 - 24**	**25 - 29**	**30 - 50**	
**Variables**	**% (n)**	**% (n)**	**% (n)**	**% (n)**	**Fisher’s exact**
**Age**					
<30 (RC)	18.4 (7)	15.8 (6)	36.8 (14)	29.0 (11)	0.45
30-59	15.7 (8)	23.5 (12)	29.4 (15)	31.4 (16)	
60-101	27.3 (3)	0.0 (0)	54.6 (6)	18.2 (2)	
**Sex**					
Male	18.0 (16)	19.1 (17)	33.7 (30)	29.2 (26)	0.89
Female	18.2 (2)	9.1 (1)	45.5 (5)	27.3 (3)	
**Education**					
Primary	17.1 (4)	13.0 (3)	21.7 (5)	47.8 (11)	
Secondary	20.0 (5)	12.0 (3)	44.0 (11)	24.0 (6)	0.39
College	50.0 (1)	0.0 (0)	50.0 (1)	0.0 (0)	
None	16.0 (8)	24.0 (12)	36.0 (18)	24.0 (12)	
**Ethnicity**					
Southern tribes	26.1 (6)	17.4 (4)	39.1 (9)	17.4 (4)	0.79
Mole-Dagomba	13.2 (5)	18.4 (7)	36.8 (14)	31.6 (12)	
Other–North tribe	18.0 (7)	18.0 (7)	30.8 (12)	33.3 (13)	
**Religion**					
Moslem	7.4 (4)	24.1 (13)	33.3 (18)	35.2 (19)	0.01
Christian	30.4 (14)	10.9 (5)	37.0 (17)	21.7 (10)	
**NHIS**					
Yes	21.5 (14)	16.9 (11)	35.4 (23)	26.2 (17)	0.60
No	11.4 (4)	20.0 (7)	34.3 (12)	34.3 (12)	

*Note*: α = 0.05; Kessler score: 0–19 = Likely to be well; 20–24 = Mild mental disorder.

25–29 = Moderate mental disorder; and 30–50 = Severe mental disorder. *Source*: Kessler et al. [11].

## Discussion

This study, which is likely the first of its kind in Ghana, explored the rates of mental distress among a select sample of incarcerated individuals in a prison in the northern part of Ghana. Overall, a majority of the sampled inmates interviewed for the study had moderate to severe mental distress scores on the Kessler classification scale in the four weeks preceding the interviews. This overall study finding is similar to the results reported in studies of mental disorders among incarcerated populations in other countries including the U.S, South Africa, and Nigeria [6,7,9,16].

The link between educational level and the proportion of inmates with moderate or severe mental distress was interesting. Nearly 70% of those with only a primary education had Kessler scores higher than 24, reflecting either moderate or severe mental distress. This finding of a link between primary education and moderate to severe mental disorder was rather surprising when compared to the lower rates of mental distress among inmates with very little or no formal education at all. A similar connection between education and mental distress was found in another study from Norway [17].

A majority of both sexes had scores in the moderate to severe mental disorder categories. Though it should be remembered that the sample of females was small, this study’s findings of 27% severe and 45% moderate mental distress among the female inmates are consistent with research results among Maryland’s and New York State’s prison inmates [6]. It is also similar to rates found among women in the general population in Iran, and what was found in the maximum security prison population in Nigeria [9,10].

Although majority of the inmates had registered for the national health insurance scheme (NHIS), almost equal number of the NHIS registrants and non-registrants had moderate mental distress. However, more of the NHIS non-registrants had psychological distress scores that classified them in the severe mental distress column than those who registered. This difference is not very surprising because the NHIS policy, which was initially introduced in Ghana in 2003, has been shown in many studies to have increased the population’s access to health care [18,19]. This study is the first known to estimate the rates of mental distress among the incarcerated population in the Tamale prison. It therefore provides the basis for further studies of the levels and determinants of mental disorders not only among individuals confined in the correctional system but in the general population as well, especially since there is no known prevalence data on mental disorders within the general population in Ghana. This study found a high prevalence of moderate to severe mental distress in the sampled population. Hopefully, these findings may encourage the Ghana Prisons Service, which has been severely underfunded, to realign its very limited resources to address the level of mental distress in the incarcerated population. It could also inform the decision makers in the prison system about the potential benefits and advantages of having more incarcerated individuals register for the National Health Insurance Scheme.

This study has some limitations. Because it was exploratory research, it involved only a moderate number of participants. Future studies of this population should enrol more diverse participants, which could help provide greater insight into why some groups scored higher on the Kessler Psychological Distress scale to be classified as having severe or moderate mental distress.

This research could also have benefitted from exploring the proportion of mental distress at multiple prison facilities, particularly comparing the Northern Region correctional facility with facilities in the southern part of the country where the cultural makeup of the prison population may be different.

Another limitation of this study is that it used only the Kessler tool to assess the respondents. Because the tool has only 10 items, what it can measure is limited. In addition, the tool only assesses broad, non-specific psychological distress, rather than specific mental disorders. It should also be noted that the K10 was not used to yield an estimate of clinical need among the study participants.

## Conclusions

Overall, the study prevalence of non-specific mental distress among the imprisoned population in Northern Ghana in this exploratory study was high. However, this finding does not indicate that the inmates at the facility with high scores on the psychological distress tool warrant any immediate clinical attention. Because the inmates with NHIS registration were found in this study to have less mental distress than those who were not registered, more could be done to increase enrollment in the national health insurance program, which is available to all eligible inmates. Study findings also demonstrate the need for significant policy changes at the national level with regard to the incarcerated population. Ghana’s prison population includes inmates who are waiting to be processed by the criminal justice system. The slow pace of the justice system means that some individuals who have only been charged with minor or petty crimes might be held for long periods of time in the over-crowded prison facilities. Regardless of why they are in prison, such incarceration could be a contributor to these high rates of distress across age groups on the Kessler scale. Ghana’s government, especially the leadership of the Ministry of Interior (with oversight of the Ghana Prisons Service), could invest in early and frequent screening of both incoming and current inmates to identify those with early signs of moderate to severe mental distress and provide them with appropriate early mental health interventions. Investing in such measures would help to ensure that emphasis is placed on screening, brief interventions, and referral to treatment (SBIRT).

Although use of the Kessler tool (K10) in this study provides the opportunity to determine the level of mental distress in one of Ghana’s many prisons, higher psychological distress scores observed in this particular prison does not imply estimates of clinical need among the inmates. Findings from this exploratory study could be incorporated as part of that correctional facility’s routine operational procedures so that current and new prison staff are aware of the high prevalence of non-specific psychological distress among inmates. Staff should be trained on warning signs of mental distress and appropriate ways to respond to inmates who may be experiencing it. The Ghana Prisons Service could also select and train some prison staff, including the prison medical staff, to operate as behavioural emergency services teams (BEST) who could supervise inmates who are identified as having mental distress.

This study can be used as baseline information to inform future research in mental distress among individuals in the correctional system. More comprehensive research should be done nationwide to determine the true prevalence of mental distress among all incarcerated individuals in the Ghana prisons system, and to help ensure that those who need it get adequate treatment.
